# Dietary carbon loaded with nano-ZnO alters the gut microbiota community to mediate bile acid metabolism and potentiate intestinal immune function in fattening beef cattle

**DOI:** 10.1186/s12917-022-03483-2

**Published:** 2022-12-06

**Authors:** Haibo Zhang, Weikun Guan, Lizhi Li, Dongsheng Guo, Xiangfei Zhang, Jiuqiang Guan, Runxiao Luo, Siying Zheng, Jiangwen Fu, Yingying Cheng, Qin He

**Affiliations:** 1 Institute of College of Life Science and Resources and Environment, Yichun University, Yi Chun, 336000 China; 2 Sichuan Academy of Grassland Sciences, Sichuan, 625014 Chengdu China; 3 College of Life Sciences, Nanchang Normal University, Nanchang, 330032 China

**Keywords:** Cattle, Carbon loaded with nano-ZnO, Gut microbiota, Bile acid metabolism, Intestinal immune function

## Abstract

**Background:**

To our knowledge, carbon loaded with nano-ZnO (NZnOC) represents a new nutritional additive for the animal husbandry industry. However, the mechanism by which NZnOC mediates beef cattle growth and intestinal health is not fully understood. This study aimed to investigate the effects of carbon loaded with nano-ZnO (NZnOC) supplementation on growth performance, gut microbiota, bile acid (BAs) metabolism and intestinal immunity in fattening cattle. Twenty cattle (16 ± 0.95 months) were randomly assigned to two dietary groups: CON (control, without feed additive) and NZnOC (diet supplemented with 80 mg NZnOC/kg diet dry matter basic) for 60 d. The colon digesta microbiota composition and BAs concentration were determined by microbiota metagenomics and gas chromatography methods, respectively.

**Results:**

The results showed that the NZnOC-supplemented cattle had greater final weight, average daily gain and gain-to-feed ratio than those in the CON group. Cattle fed the NZnOC diet had a higher relative abundance of the secondary BAs synthesizing phyla Firmicutes, *Tenericutes* and *Actinobacteria* than those fed the CON diet. Dietary supplementation with NZnOC increased the relative abundance of the secondary BAs synthesis microbiota genera *Clostridium*, *Ruminococcus*, *Eubacterium*, and *Brevibacillus* in colon digesta. Cattle fed the NZnOC diet had increased activities of 3α-hydroxysteroid dehydrogenase (EC: 1.1.1.52) and bile acid-CoA ligase BaiB (EC: 6.2.1.7) in the colon digesta compared with those fed the CON diet. The primary BAs taurocholic acid, taurochenodeoxycholic acid and taurodeoxycholate acid were significantly decreased by dietary NZnOC supplementation, while the secondary BAs deoxycholic acid, taurolithocholic acid, beta-muricholic acid, 12-ketolithocholic acid and ursodeoxycholic acid were significantly increased. Dietary supplementation with NZnOC increased the mRNA abundance of G protein-coupled bile acid receptor 1, protein kinase cAMP-activated catalytic subunit alpha, cyclic-AMP response element binding protein 1 and interleukin (*IL*)-10 in the colon mucosa of cattle, while the mRNA abundance of tumor necrosis factor and IL-1β were significantly decreased.

**Conclusions:**

In summary, dietary supplementation with NZnOC can facilitate the growth performance and intestinal immune function of cattle by improving BAs metabolism. NZnOC can be supplemented in the diet as a safe regulator of gut microbiota and as a feed additive in the ruminants industry.

## Background

Several nutritional factors can affect beef cattle production, such as the roughage vs. concentrate ratio, glucose/starch availability, dietary energy and protein levels, and stage-specific feeding systems [1, 2]. A growth stage-specific feeding system has generally been applied to produce highly marbled beef. The fattening period can be divided into early and late periods. During the late fattening phase (2–5 months), concentrate is mainly fed with a small amount of roughage [2]. In intensive beef feeding systems such as feedlots also in the diet used more rapid fermentation carbohydrates (approx. 35–36% dry matter (DM) starch and lower level of neutral detergent fiber approx. 28–32% DM). Cattle are frequently fed high-concentrate diets in modern intensive feeding diets, which may disrupt the ruminal microbiota balance and lead to subacute ruminal acidosis (SARA) [3]. Although the etiology and pathophysiology of SARA have been studied, there is no clear definition of the condition [4–6]. A new precise definition related to the presence of lipopolysaccharides (LPS) in the systemic circulation may involve LPS receptors affecting leukocyte populations and triggering the production of proinflammatory cytokines and acute phase proteins [6]. Carbon loaded with nano-ZnO (NZnOC) represents a new nutritional additive for the food and animal husbandry industry. In the current study NZnOC with premix was provided and was not encapsulated, so might effect also on the ruminal microbiota. NZnOC/kg to the total mixed ration (TMR) might positively affect the ruminal microbiota, decrease an LPS concentration, and protect against hepatic and intestine inflammation.

Intestinal inflammation damages the intestinal microbiota of cattle, resulting in diarrhea and bloody stool, weight loss, and death in severe cases [7, 8]. Previous studies have shown that intestinal inflammation is the result of the interaction of factors such as the environment, genetic susceptibility and intestinal microbiota [9]. Bile acids (BAs) are metabolized by intestinal microbiota to produce secondary BAs, which can regulate glucose and lipid metabolism and immune function in animals [10–12]. BAs combined with G protein-coupled bile acid receptor 1 (GPBAR1) enhances *GPBAR1* gene expression and upregulates the gene expression of the protein kinase cAMP-activated catalytic subunit alpha (*PRKACA*) and cyclic-AMP response element binding protein 1 (*CREB1*), reducing the expression and secretion of proinflammatory cytokines such as TNF and IL-1β and increasing the expression and secretion of anti-inflammatory cytokines such as IL-10 [13, 14]. The above studies show that the intestinal microbiota-BAs-GPBAR1-PRKACA-CREB1 signaling pathway regulates host BAs metabolism, alleviates the inflammatory response and enhances immune function. The discovery of this new pathway provides a new theoretical basis and direction for the research and development of nutritional additives that regulate the structure of intestinal microbiota, enhance immune function and alleviate animal intestinal inflammation.

Previous studies have shown that dietary supplementation with nano zinc oxide (NZnO) increased the relative abundance of the secondary BAs synthesizing genera *Lactobacillaceae*, *Firmicutes* and *Lactobacillus* in pigs at the colon [15]. At the same time, many studies have shown that NZnO improves intestinal immunity function [16, 17]. Dietary supplementation with 800 mg/kg NZnO significantly increased anti-inflammation (IL)-10 cytokines and inflammation (*TNF* and *IL-1β*) mRNA expression in pigs [17]. Hu et al. demonstrated that NZnO-supplemented pigs had lower *TNF*, *IFN-γ* and *IL-1β* mRNA expression levels in the ileal mucosa [16]. These results suggested that NZnO may regulate the intestinal immune function of pigs by modulating intestinal BAs metabolism-related microbiota. In recent years, taking carbon as the carrier of trace elements to supplement the trace element zinc required by the body through dietary intake has become a new method of zinc supplementation [18, 19]. Compared with zinc carriers prepared by traditional methods, NZnOC has the advantages of a simple preparation process, low cost and mass production. China’s Ministry of Agriculture and Rural Affairs (MARA) issued No. 2625 recommendation that the use of NZnOC should not exceed 120 mg/kg zinc diet DM in the nutrition of ruminants. However, the application of NZnOC in cattle breeding has not yet been reported. In addition, the primary BAs is bioconverted to secondary BAs by intestinal biological modification (deconjugation, dehydroxylation and epimerization) [20–23]. Thus, whether NZnOC as an animal feed additive can enhance secondary BAs synthesis microbiota growth and promote secondary BAs synthesis through biological modification (deconjugation, dehydroxylation and epimerization) and facilitate immune function requires further exploration.

Therefore, we hypothesize that NZnOC (our own synthetic) alters the growth performance and gut microbiota community to mediate BAs metabolism and enhance intestinal immune function by the secondary BAs-GPBAR1-PRKACA-CREB1 signaling pathway of fattening cattle. The objective of our study was to investigate the effects of NZnOC supplementation on growth performance, gut microbiota, BAs metabolism and intestinal immunity in fattening beef cattle. The data obtained might provide a theoretical basis for the rational use of new zinc supplementation in cattle production, which would be useful in improving intestinal immune function and promoting cattle health.

## Results

### Growth performance

As shown in Table 1, the NZnOC-supplemented cattle had greater final weight, average daily gain (ADG) and gain-to-feed ratio than those in the CON group (*p* < 0.05). No significant differences in initial weight or total DM intake (TDMI) were observed between the two groups.

**Table 1:** Effects of dietary NZnOC supplementation on growth performance of fattening cattle (average values per animal)

Item	CON group	NZnOC group
Initial BW, kg	409.88 ± 10.24	413.54 ± 10.81
Final BW, kg	466.76 ± 13.33^b^	486.30 ± 5.76^a^
ADG, kg/day	0.95 ± 0.07^b^	1.17 ± 0.15^a^
TDMI, kg	8.17 ± 0.30	8.30 ± 0.42
Gain-to-feed ratio	0.116 ± 0.0095^b^	0.143 ± 0.0150^a^

*CON* Control, *NZnOC* Carbon loaded with nano-ZnO, *BW* Body weight, *TDMI* Total dry matter intake, *ADG* Average daily gain

Cattle were regarded as the experimental units, *n* = 10/group. Within a row, values with different superscripts indicate a significant difference (*p* < 0.05)

### Colonic digesta microbiota composition at the phylum and genus levels

**Fig. 1:**
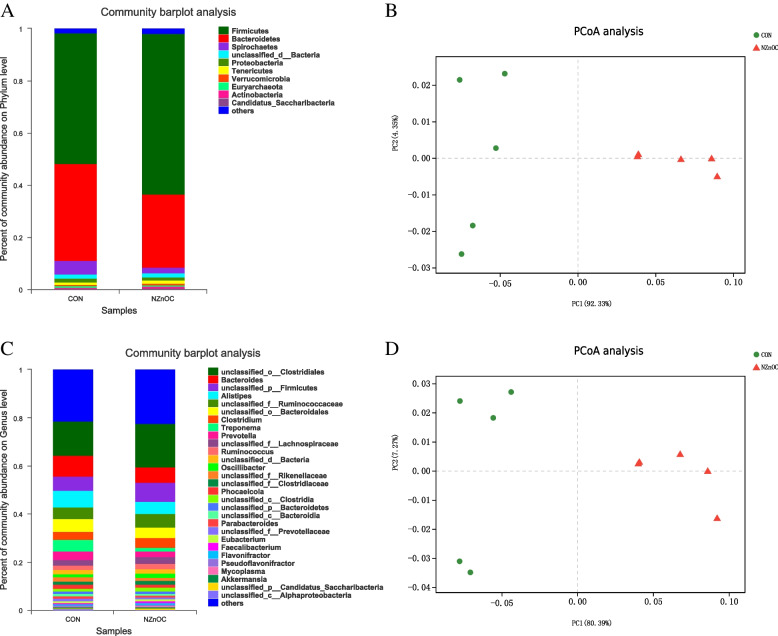
**A-D** Effect of NZnOC on colon chyme microbiota diversity. **A** phylum level, (**B**) PCoA analysis at phylum level, (**C**) genus level, (**D**) PCoA analysis at genus level. CON, control; NZnOC, carbon loaded with nano-ZnO (NZnOC); PCoA, principal co-ordinates analysis. Cattle were regarded as the experimental units, *n* = 5/group

The relative abundance of bacteria at the phylum level is presented in Fig. 1A. The dominant phylum was Firmicutes (50.07–61.70%), followed by Bacteroidetes (28.10–37.06%) in all groups. Principal coordinate analysis (PCoA) results showed that the CON group and NZnOC group microbiota were clustered separately, and their axes accounted for 96.68%, indicating that some bacterial species may be affected by NZnOC (Fig. 1B).

Effects of dietary NZnOC supplementation on the relative abundance of bacteria at the genus level (Fig. 1C). In the CON group, the top 4 dominant genera were *unclassified_o_Clostridiales* (14.21%), *Bacteroides* (8.62%), *Alistipes* (6.92%), and *unclassified_p_Firmicutes* (6.00%) (Fig. 1C). In the NZnOC group, the top 4 dominant genera were *unclassified_o_Clostridiales* (18.24%), *unclassified_p_Firmicutes* (7.93%), *Bacteroides* (6.24%), and *unclassified_f_Ruminococcaceae* (5.61%) (Fig. 1C). At the genus level, the PCoA results showed that the microbiota clustered separately, and their axes accounted for 97.66%, suggesting that certain key bacterial species may characterize the microbiota of the NZnOC group (Fig. 1D).

### Differential colonic digesta microbiota communities

**Fig. 2:**
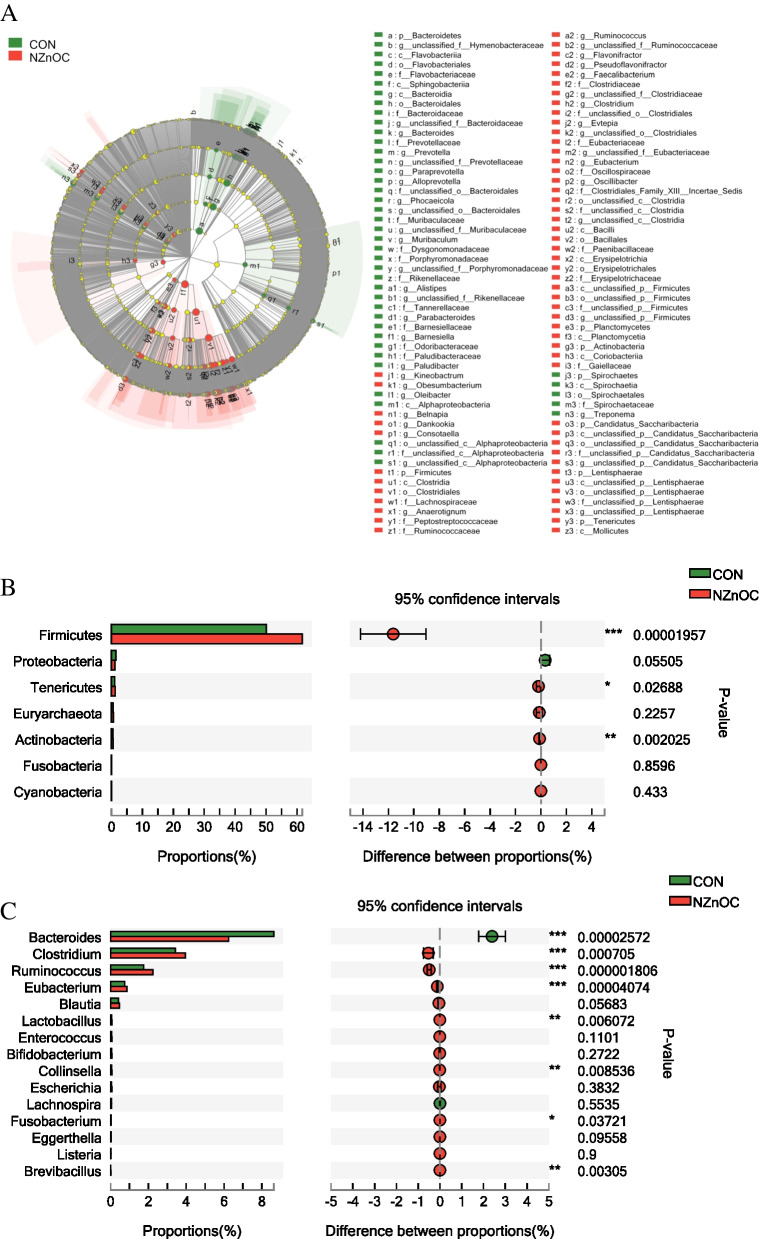
**A-C** Differential colonic digesta microbiota communities change between two groups. **A** LEfSe analysis displaying on bacterial community, (**B**) Welch’s t-test on secondary BA synthesis microbiota at phylum, (**C**) Welch’s t-test on secondary BA synthesis microbiota at genus. LDA ≥ 2.5 and *P* ≤ 0.05 were shown. CON, control; NZnOC, carbon loaded with nano-ZnO; BA, bile acid; LDA, linear discriminant analysis; p_, phylum; c_, class; o_, order; f_, family; g_, genus. Cattle were regarded as the experimental units, *n* = 5/group. *p < 0.05, **p < 0.01, ***p < 0.001

To identify the taxon that had a great impact on the microbiota community, LEfSe analysis was performed, and 104 biomarkers were found in the CON and NZnOC groups (Fig. 2A). The cattle fed NZnOC had significantly increased abundances of the genera *unclassified_o__Clostridiales*, *Obesumbacterium*, *unclassified_p__Firmicutes*, *unclassified_c__Clostridia*, *Evtepia*, *unclassified_p__Candidatus_Saccharibacteria*, *Dankookia*, *Kineobactrum*, *unclassified_f__Clostridiaceae*, *Anaerotignum*, *Pseudoflavonifractor*, *unclassified_f__Ruminococcaceae*, *unclassified_p__Lentisphaerae*, *unclassified_f__Eubacteriaceae*, *Consotaella*, *Belnapia*, *Oscillibacter*, *Flavonifractor*, *Eubacterium*, *Clostridium*, *Ruminococcus*, and *Faecalibacterium* and the phyla *Firmicutes*, *Actinobacteria*, *Planctomycetes*, *Candidatus_Saccharibacteria*, and *Tenericutes* (*p* < 0.05).

Welch’s t test was used to analyse secondary BAs synthesis microbiota between the two groups. As shown in Fig. 2B, cattle fed the NZnOC diet had significantly higher relative abundances of the phyla *Firmicutes*, *Tenericutes* and *Actinobacteria* than those fed the CON diet (*p* < 0.05). At the genus level (Fig. 2C), dietary supplementation with NZnOC significantly increased the abundance of *Clostridium* (*p* = 0.000705), *Ruminococcus* (*p* = 0.000001806), *Eubacterium* (*p* = 0.00004074), *Lactobacillus* (*p* = 0.006072), *Lachnospira* (*p* = 0.003721), *Collinsella* (*p* = 0.008536), *Fusobacterium* (*p* = 0.03721) and *Brevibacillus* (*p* = 0.00305) in the colon digesta compared with the CON group. *Bacteroides* (*p* = 0.00002572) in the NZnOC group were significantly lower than those in the CON group.

### Secondary BAs synthesis pathway analysis

**Fig. 3:**
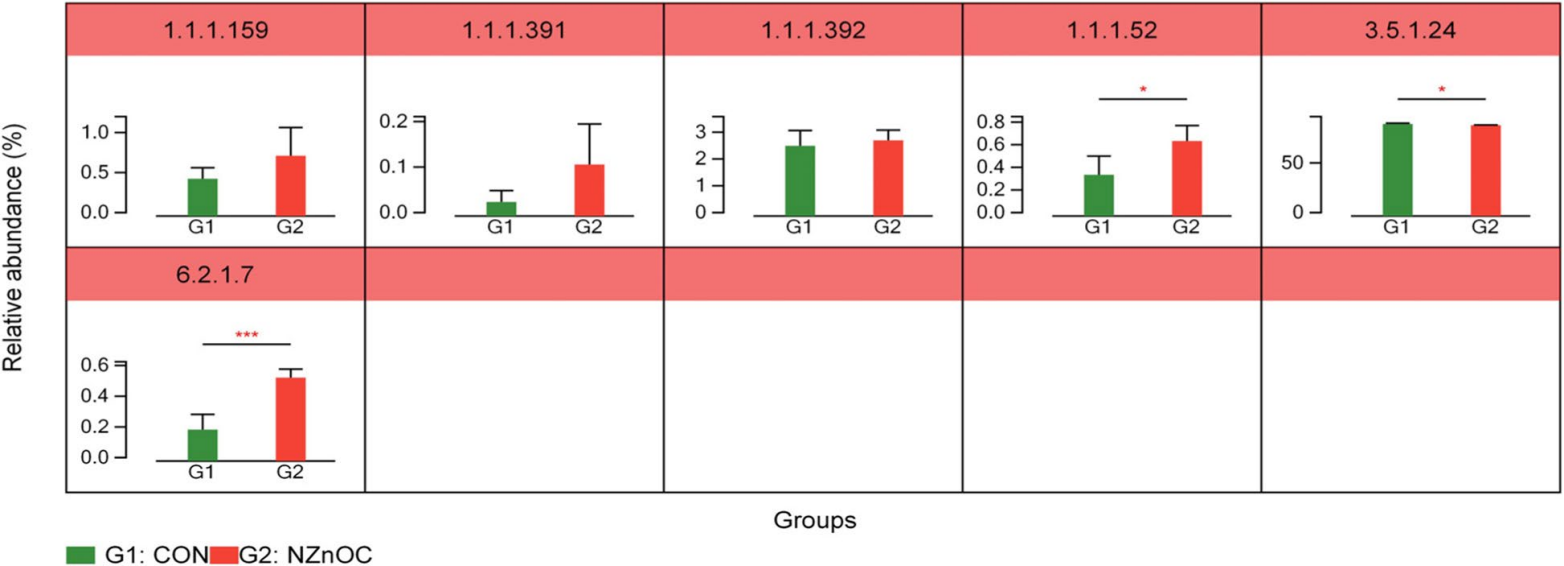
Secondary BA synthesis pathway analysis displaying the change between two groups. **A** differ secondary BA synthesis pathway, (**B**) differ enzyme. CON, control; NZnOC, carbon loaded with nano-ZnO. Cattle were regarded as the experimental units, *n* = 5/group. *p < 0.05, ***p < 0.001

The ko00121 pathway is a secondary BAs synthesis pathway according to the KEGG pathway database information. Welch’s T test was used to analyse the secondary BAs synthesis pathway between the two groups (Fig. 3). As shown in Fig. 3, cattle fed the NZnOC diet had markedly increased activities of 3α-hydroxysteroid dehydrogenase (EC: 1.1.1.52) and bile acid-CoA ligase BaiB (EC: 6.2.1.7) in the colon digesta compared with those fed the CON diet (*p* < 0.05).

### BAs content analysis

**Fig. 4:**
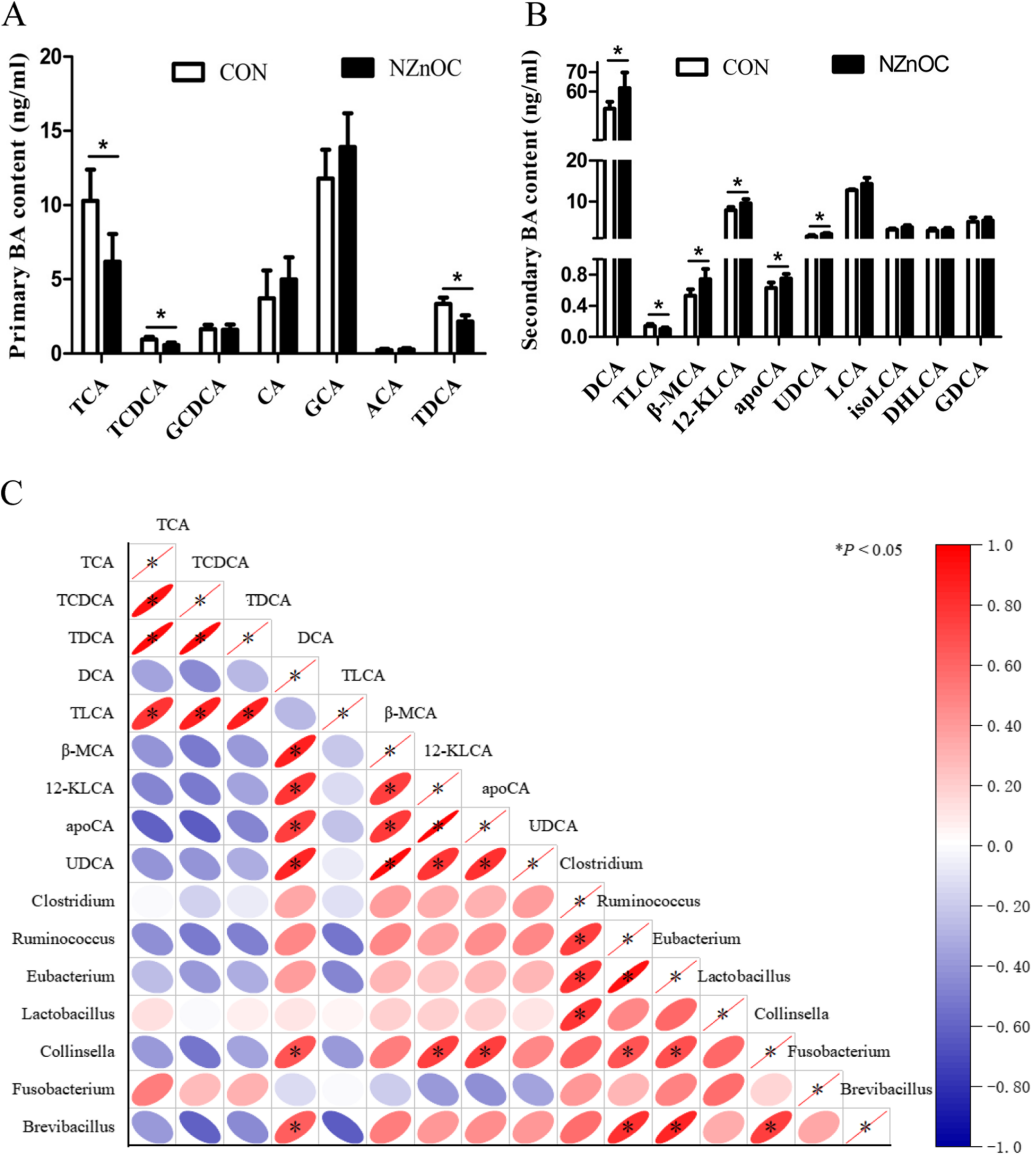
**A-C** Effects of dietary NZnOC supplementation on BA content analysis. **A** Primary BA content, (**B**) Secondary BA content, (**C**) Spearman correlation analysis between secondary BA synthesis bacterial genera and secondary BA content. Means ± SD, *n* = 5. Values with different *asterisks indicate signifificant (*P* < 0.05). The positive correlation (closer to 1) for red and the negative correlation (closer to − 1) for blue. CON, control; NZnOC, carbon loaded with nano-ZnO; TCA, taurocholic acid; TCDCA, taurochenodeoxycholic acid; GCDCA, glycochenodeoxycholic acid; CA, cholic acid; GCA, glycocholic acid; ACA, allocholic acid; TDCA, taurodeoxycholate acid; DCA, deoxycholic acid; TLCA, taurolithocholic acid; β-MCA, beta-muricholic acid; 12-KLCA, 12-ketolithocholic acid; apoCA, apocholic acid; UDCA, ursodeoxycholic acid; LCA, lithocholic acid; isoLCA, isolithocholic acid; DHLCA, dehydrolithocholic acid; GDCA, glycodeoxycholic acid. *p < 0.05

The primary BAs taurocholic acid (TCA), taurochenodeoxycholic acid (TCDCA) and taurodeoxycholate acid (TDCA) were significantly decreased by dietary NZnOC supplementation (*p* < 0.05; Fig. 4A), while the secondary BAs deoxycholic acid (DCA), taurolithocholic acid (TLCA), beta-muricholic acid (β-MCA), 12-ketolithocholic acid (12-KLCA), apocholic acid (apoCA) and ursodeoxycholic acid (UDCA) were significantly increased (*p* < 0.05; Fig. 4B). However, there were no significant differences in glycochenodeoxycholic acid (GCDCA), cholic acid (CA), glycocholic acid (GCA), allocholic acid (ACA), lithocholic acid (LCA), isolithocholic acid (isoLCA), dehydrolithocholic acid (DHLCA) or glycodeoxycholic acid (GDCA) between the two groups (*p* > 0.05; Fig. 4A, B).

To investigate the connection between the secondary BAs concentration and the microbiota community, we compared the taxa revealed by LEfSe analysis with the secondary BAs content. The correlation matrixes were created according to the relative abundance of secondary BAs synthesis bacterial genera and the secondary BAs content (Fig. 4C). DCA was significantly positively correlated with the genera *Collinsella* and *Brevibacillus* (*p* < 0.05). 12-KLCA and apoCA were significantly positively correlated with the genus *Collinsella* (*p* < 0.05).

### COG Orthology analysis

**Fig. 5:**
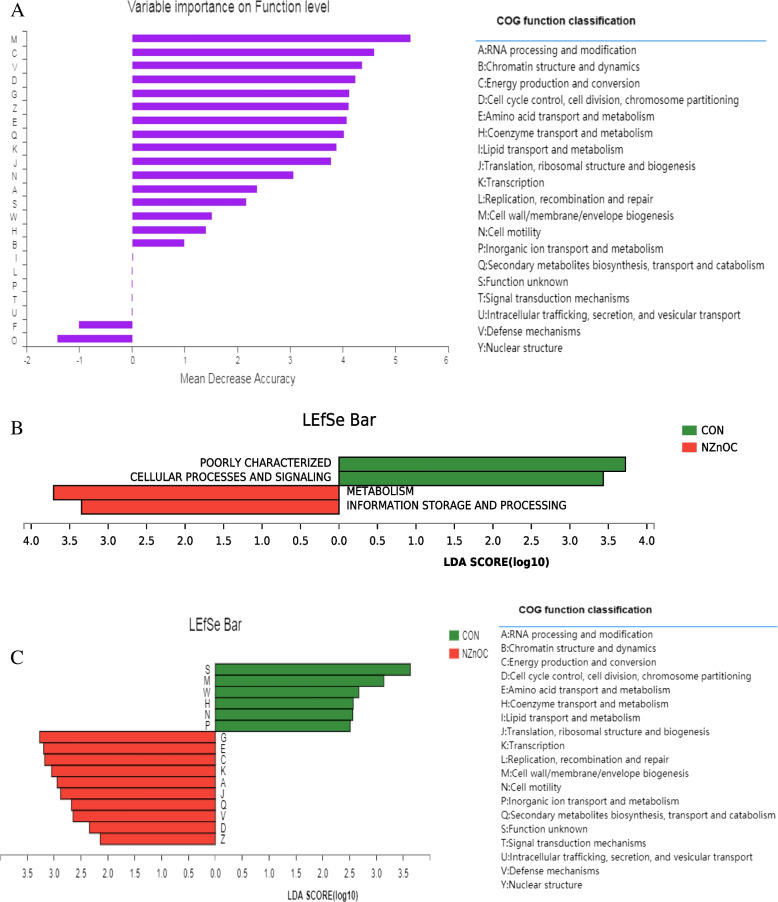
**A-C** COG Orthology analysis displaying the change between two groups. **A** functional importance ranking by random forest analysis, (**B**) differ category by LEfSe analysis, (**C**) differ function by LEfSe analysis. CON, control; NZnOC, carbon loaded with nano-ZnO. Cattle were regarded as the experimental units, *n* = 5/group

According to the eggNOG database, the top 5 functions, cell wall/membrane/envelope biogenesis (M), energy production and conversion (C), defense mechanisms (V), chromosome p (D), carbohydrate transport and metabolism (G), were found by using the random forest method (Fig. 5A).

Welch’s t test was used to analyse the categories and functions between the two groups by COG abundance of the top 30. As shown in Fig. 5B, dietary NZnOC supplementation significantly increased the abundance of genes in the category of metabolism, information storage and processing and reduced the abundance of genes in the category of poorly characterized cellular processes and signaling in the colon of cattle (*p* < 0.05). Moreover, compared with the CON group, NZnOC-supplemented cattle had higher carbohydrate transport and metabolism (G), amino acid transport and metabolism (E), energy production and conversion (C), transcription (K), RNA processing and modification (A), translation, ribosomal structure and biogenesis (J), secondary metabolites biosynthesis, transport (Q), defense mechanisms (V), chromosome p (D), and cytoskeleton (Z) at the functional level in the colon digesta (*p* < 0.05; Fig. 5C).

### Colon mucosa immune function analysis

**Fig. 6:**
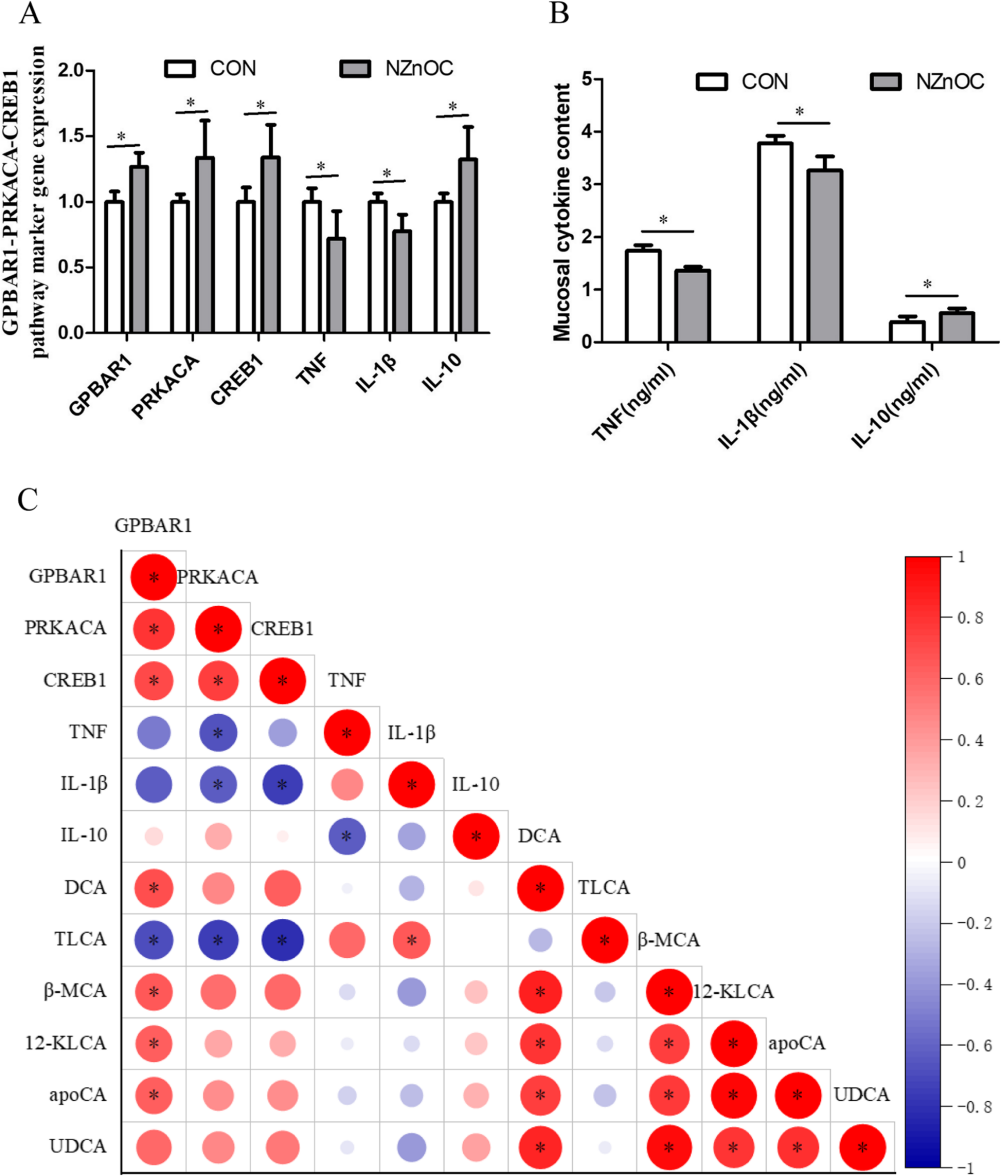
**A-C** Effect of dietary NZnOC supplementation on intestinal immune function of cattle. **A** GPBAR1-PRKACA-CREB1 pathway marker gene expression, (**B**) Colon mucosal cytokine content, (**C**) Spearman correlation analysis between GPBAR1-PRKACA-CREB1 pathway marker gene expression and secondary BA content. Means ± SD, *n* = 5. Values with different *asterisks indicate signifificant (*P* < 0.05). CON, control; NZnOC, carbon loaded with nano-ZnO; GPBAR1, G protein-coupled bile acid receptor 1; PRKACA, protein kinase cAMP-activated catalytic subunit alpha; CREB1, cyclic-AMP response element binding protein 1; TNF, tumor necrosis factor; IL-1β, interleukin-1 β; IL-10, interleukin-10; DCA, deoxycholic acid; TLCA, taurolithocholic acid; β-MCA, beta-muricholic acid; 12-KLCA, 12-ketolithocholic acid; apoCA, apocholic acid; UDCA, ursodeoxycholic acid; apoCA, apocholic acid. *p < 0.05

As shown in Fig. 6, dietary supplementation with NZnOC increased the mRNA abundance of *GPBAR1*, *PRKACA*, *CREB1* and *IL-10* in the colon mucosa of cattle, while the mRNA abundance of *TNF* and *IL-1β* was significantly decreased (*p* < 0.05; Fig. 6A). Compared with the CON group, cattle in the NZnOC group had higher mucosal IL-10 content and lower TNF and IL-1β contents (*p* < 0.05; Fig. 6B).

Spearman nonparametric rank correlation analysis between GPBAR1-PRKACA-CREB1 pathway marker gene expression and secondary BAs content is presented in Fig. 6C. The *GPBAR1* gene expression was significantly positively correlated with DCA, β-MCA, 12-KLCA and apoCA (*p* < 0.05). The *GPBAR1* gene expression was significantly positively correlated with the *PRKACA* and *CREB1* gene expression (*p* < 0.05). *IL-1β* gene expression was significantly positively correlated with TLCA (*p* < 0.05). *PRKACA* and *CREB1* gene expression was significantly negatively correlated with TLCA (*p* < 0.05).

## Discussion

In the present study, cattle gained 220 g/d in the NZnOC group more than those in the control group, which is consistent with the findings of Chang et al. (2020) [24], who showed that calves gained 120.36 g/d in receiving 80 mg of zinc from zinc-Met and 53.93 g/d in receiving 80 mg of zinc from zinc oxide. Feldmann et al. (2019) also found that zinc-Met-treated bull calves had a 22 g/d higher ADG than placebo-treated bull calves [25]. Notably, the gain-to-feed ratio in the NZnOC group was greater than that in the control group, which might be due to the higher bioavailability of NZnOC. Supplementing finishing heifers up to 60 mg zinc/kg diet DM (111.4 mg total zinc/kg diet DM) improved the gain-to-feed ratio compared to control treatments [26]. Heifers supplemented with zinc had an improved gain-to-feed ratio compared to heifers supplemented with 0 zinc (*p* = 0.04), suggesting that finishing cattle require increased concentrations of zinc than the 30 mg/kg zinc recommended by NRC (2000) for beef cattle [26, 27]. Carbon has a large unique globular surface, which makes it an excellent carrier to support nanoparticles. The NZnOC compound was formed with carbon as the carrier of NZnO, and it may therefore have the dual effects of carbon and NZnO. The results of this experiment may be due to the carbon increasing the proportion of NZnO reaching the intestine, promoting NZnO absorption and improving production performance. To clarify the mechanism of the health-promoting effect of dietary NZnOC, the metabolic and intestinal responses of cattle to the NZnOC diets were further investigated.

The gut microbiota community is highly related to host metabolic capacity and health. The gastrointestinal tract is a complex microbiota community dominated by two bacterial phyla, Bacteroidetes and Firmicutes [28, 29]. The majority of bile salt hydrolase (BSH)-expressing bacteria are members of the Firmicutes phylum, which mediates BAs biological modification [30]. Therefore, an increase in the abundance of the Firmicutes phylum promotes the conversion of primary BAs into secondary BAs, leading to decreased levels of conjugated primary BAs in the terminal ileum [31]. In this study, analysis of colon digesta by microbiota metagenomics technology demonstrated that the abundance of the phylum Firmicutes in the gut microbiota increased in cattle that received NZnOC treatment, which suggests that NZnOC may be involved in the secondary BAs synthesis process. Next, we focused on the secondary BAs synthesis microbiota at the phylum level in the cattle fed NZnOC. Previous studies have shown that the phyla *Tenericutes* and *Actinobacteria* participate in secondary BAs synthesis, which oxidizes the hydroxyl groups at positions 3, 7 or 12 of the ring chain [30, 32]. Meanwhile, we also found that the NZnOC-supplemented cattle had higher phyla *Tenericutes* and *Actinobacteria* than those in the CON group. All these data suggest that NZnOC can mediate the biological modification of BAs in cattle, but its specific mechanism needs to be further studied.

The intestinal microbiota plays a key role in regulating BAs metabolism through a series of enzyme reactions, promoting the transformation of primary BAs into secondary BAs. These transformations include deconjugation, dehydroxylation and epimerization. First, deconjugation was carried out on the side chain by microbiota BSH, an enzyme mainly expressed by anaerobic intestinal bacteria of the genera *Brevibacillus* and *Clostridium* [20–23]. BSH deconjugates primary BAs, such as TCA, TCDCA and Tβ-MCA, to form CA, chenodeoxycholic acid (CDCA) and β-MCA [10]. The current study showed that the primary BAs of TCA, TCDCA and TDCA decreases with dietary NZnOC supplementation, indicating that NZnOC may promote primary BAs conversion to secondary BAs. Then, dehydroxylation of deconjugated BAs (such as CDCA and CA) is contributed by a bacterial 7α-dehydroxylase mainly expressed by *Eubacterium*, *Clostridium* and *Ruminococcus*, which converts CA to DCA and CDCA to LCA [21]. CDCA was converted to 12-KLCA by 7α-dehydroxylation [33]. The Firmicutes phylum (*Clostridium* and *Eubacterium*) has 7α-dehydroxylation activity encoded by the BAs-inducible (baiB) gene [34]. The baiB (EC: 6.2.1.7) enzyme is involved in the 7α-de hydroxylation of the secondary BAs synthesis pathway [35]. In this experiment, NZnOC-supplemented cattle increased secondary BAs (DCA, β-MCA and 12-KLCA), which may also explain the increased relative abundance of secondary BAs-producing *Eubacterium*, *Clostridium* and *Ruminococcus* by raising bile acid-CoA ligase BaiB (EC: 6.2.1.7) abundance in the colon digesta. Spearman’s correlation confirmed that DCA was significantly positively correlated with the genus *Brevibacillus*. Taken together, these data indicate that cattle fed the NZnOC diet had an increased relative abundance of secondary BAs microbiota (*Brevibacillus*, *Eubacterium*, *Clostridium* and *Ruminococcus*), which could play an accelerative secondary BAs (DCA, β-MCA and 12-KLCA) production role in the BAs deconjugation and dehydroxylation processes.

Other bacterial genera contribute to different BAs epimerization metabolism: *Eubacterium*, *Clostridium*, *Eubacterium*, *Ruminococcus*, *Lactobacillus*, and *Collinsella*, which catalyze oxidation and epimerization by the hydroxylsteroid dehydrogenase family; *Eubacterium* might carry out BAs esterification reactions, while *Fusobacterium* and *Clostridium* are able to desulfate BAs [20, 21]. In this experiment, we found that dietary supplementation with NZnOC increased the secondary BAs-producing genera *Clostridium*, *Ruminococcus*, *Eubacterium*, *Lachnospira*, *Collinsella*, *Fusobacterium* and *Brevibacillus* in the colon digesta, which suggested that NZnOC may function through BAs epimerization metabolism in secondary BAs synthesis. Previous studies demonstrated that CDCA generated DCA and UDCA by 7α/β-epimerization, and αMCA generated β-MCA at the 7α-hydroxyl group transforms [36]. UDCA is converted to LCA by 7α/β-dehydroxylase, and TLCA is formed from LCA [36, 37]. In our study, we also found that NZnOC-supplemented cattle increased secondary BAs of DCA, TLCA and UDCA contents, which may explain the increased relative abundance of secondary BAs-producing *Clostridium*, *Ruminococcus*, *Eubacterium*, *Lachnospira*, *Collinsella*, *Fusobacterium* and *Brevibacillus* in the colon digesta. Next, to investigate the secondary BAs synthesis pathway in BAs epimerization metabolism, we compared the ko00121 pathway of different enzyme activities revealed at BAs epimerization by the Welch T test. Current results showed that cattle fed the NZnOC diet had markedly increased activities of 3α-hydroxysteroid dehydrogenase (EC: 1.1.1.52). In summary, cattle fed the NZnOC diet increased secondary BAs (DCA, TLCA and UDCA) by promoting the secondary BAs-producing genera *Clostridium*, *Ruminococcus*, *Eubacterium*, *Lachnospira*, *Collinsella*, *Fusobacterium* and *Brevibacillus* by increasing 3α-hydroxysteroid dehydrogenase (EC: 1.1.1.52) abundance during the BAs epimerization processes.

Random forest is an integrated learning method that includes multiple decision trees for classification, regression or other tasks. Random forest is often used to predict the important characteristic species, functions, genes or other attributes that affect the overall distribution of the community, which is helpful to further reveal the great biological significance hidden behind the data. In this experiment, we found that the top 3 functions were cell wall/membrane/envelope biogenesis (M), energy production and conversion (C), and defense mechanisms (V) by using random forest methods. The results showed that one of the important functions of microbiota was defense mechanisms (V). Next, to identify COG orthology analysis, LEfSe analysis was performed, and differentially abundant taxa were found in both groups. Our data showed that NZnOC increased the abundance of genes involved in metabolism and increased defense mechanisms at the category and functional levels, suggesting that NZnOC may enhance the intestinal mucosal immunity of cattle.

The current study showed that the secondary BAs of DCA, TLCA and UDCA contents were significantly increased by dietary NZnOC supplementation. We also found that dietary supplementation with NZnOC increased the mRNA abundance of *GPBAR1*, *PRKACA* and *CREB1* in the colon mucosa of cattle. Pearson’s correlation also confirmed that *GPBAR1* gene expression was significantly positively correlated with *PRKACA* and *CREB1* gene expression; *GPBAR1* gene expression was significantly positively correlated with DCA, β-MCA and 12-KLCA. Previous studies showed that DCA, TLCA and UDCA could directly act on intestinal epithelial GPBAR1 mRNA expression to upregulate the mRNA abundance of PRKACA and CREB1 [38, 39]. All these data suggested that NZnOC mediates cattle intestinal mucosal immunity via the GPBAR1-PRKACA-CREB1 signaling pathway. The GPBAR1-PRKACA-CREB1 signaling pathway could decrease the expression and secretion of proinflammatory cytokines such as TNF and IL-1β and increase the expression and secretion of anti-inflammatory cytokines such as IL-10 [7, 8]. Noticeably, our findings showed that NZnOC increased the expression and secretion of anti-inflammatory cytokines (IL-10) and reduced the expression and secretion of proinflammatory cytokines (TNF and IL-1β). These results indicated that NZnOC modulated cattle’s intestinal mucosal immunity by regulating the intestinal microbiota-BAs-PRKACA-CREB1 signaling pathway.

## Conclusion

In conclusion, our results demonstrated a potential beneficial role of NZnOC in improving growth performance and intestinal health in beef cattle, possibly via mechanisms associated with enhancing secondary BAs synthesis microbiota growth and secondary BAs synthesis by microbiota biological modification, modulating the secondary BAs-PRKACA-CREB1 signaling pathway, and changing the immune status. These findings indicate the potential application of NZnOC as a safe and effective nutritional intervention strategy for maintaining gut health in mammals.

## Methods

### NZnOC synthesis

**Fig. 7:**
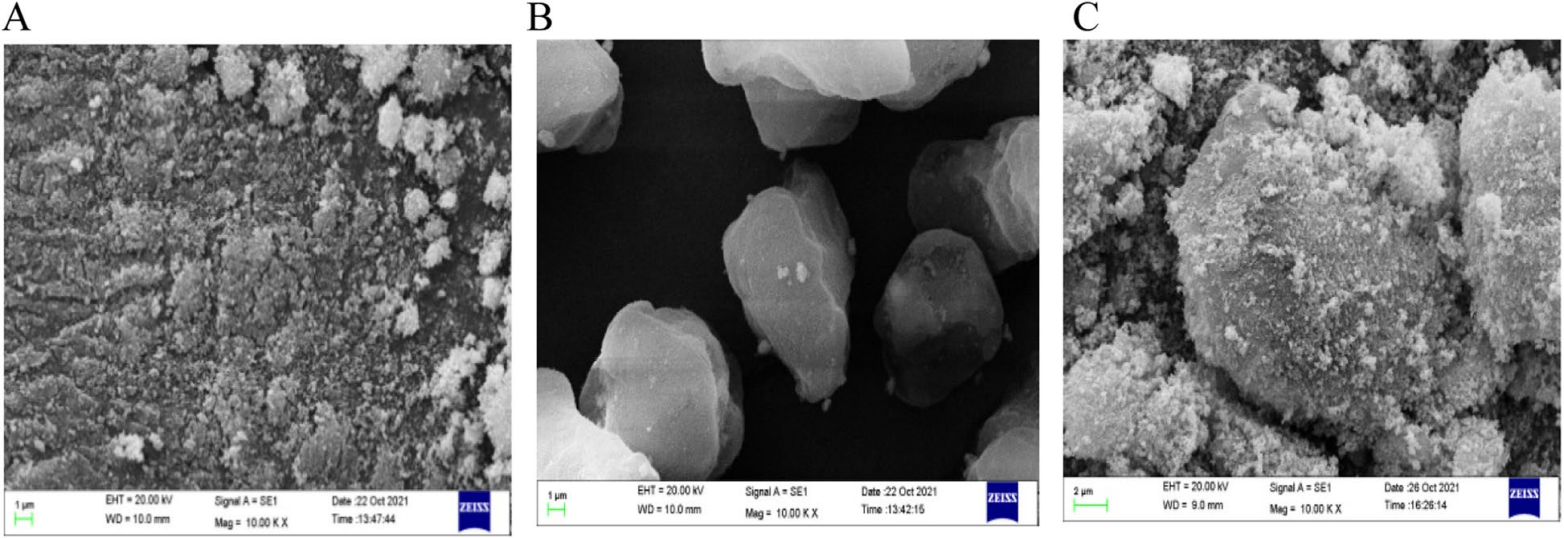
**A-C** Morphologies of NZnO, C and NZnOC. **A** Nano-ZnO (NZnO), (**B**) Carbon (**C**), (**C**) Carbon loaded with nano-ZnO (NZnOC)

In the first step, 10 mg carbon and 400 mg NZnO were dissolved and dispersed into 20 ml dimethylformamide (DMF) solution, respectively, and ultrasonic stirring was performed for 30 min. Then, NZnO dissolved in DMF was added droplet by droplet to the DMF dispersion of carbon. The solution was stirred and ultrasonicated for 15 min to obtain a uniform and precipitation-free liquid. The liquid was then sealed into a 50 ml hydrothermal synthesis reactor with a Teflon lining and placed into an electric blast drying oven at 220 °C for 3 h of reaction. The products produced in this step are separated by centrifugation and cleaned several times with deionized water. In the second step, the products produced in the first step were fully dispersed into 35 ml deionized water, encapsulated in a hydrothermal synthesis reaction kettle with a Teflon lining, and then placed in an electric blast drying oven at 180 °C for 21 h of reaction. The obtained product was separated by extraction, filtered and washed with deionized water and anhydrous ethanol several times. The final product was dried for 24 h at 80 °C in an electric vacuum drying oven. The loading of NZnO (Fig. 7A) onto the carbon (Fig. 7B) surface does not change the spherical structure of carbon, and NZnO is evenly distributed on the surface of carbon to form the NZnOC composite (Fig. 7C).

### Animals, experimental design, diets, housing, growth study and sample collection

The experiment was conducted in Chengdu from May to July 2021. A total of 20 healthy beef cattle (Simmental × Chinese Yellow Cattle, 16 ± 0.95 months, male, and body weight: 412.71 ± 10.91 kg) were randomly assigned by body weight to one of two dietary treatments (*n* = 10), consisting of the basal diet (CON) or the basal diet supplemented added with 80 mg NZnOC/kg diet DM as a treatment. The animals were obtained from the same farm (Xing Cezheng beef cattle farm) of Chengdu, China. Cattle were physically examined by a veterinarian. The health status of beef cattle was good physical development, reasonable body structure, excellent mental status, smooth skin, smooth coat, and no symptoms of disease. The 80 mg NZnOC/kg diet DM was equivalent to 60.58 mg zinc/kg diet DM. The experimental dose of NZnOC is associated with dairy cow, calf and feedlot heifer experiments and uses zinc prepared by traditional methods or zinc oxide [24–26]. This level of zinc supplementation was determined on the basis of previous publications [26]. Feedlot heifers supplemented with up to 60 mg zinc/kg diet DM (111.4 mg/kg total dietary zinc) had the greatest improvement in feed efficiency with minimal effects on carcass characteristics [26]. The adaptation period was 7 days, and the experimental period lasted for 60 days. The experimental period was determined based on the timing of short-term fattening in beef cattle [1, 2].

The nutritional level of the diet refers to the feeding standard for beef cattle, considering an ADG of 1 kg [40]. Vitamin-mineral premixes were formulated to provide either 0 or 60.58 mg supplemental zinc/kg diet DM as the NZnOC form (80 mg NZnOC/kg diet DM). The vitamin-mineral premixes were weighed daily and subsequently mixed into total mixed rations. The test diets were fully mixed by a TMR feed mixer. The composition and nutritional level of the basal diet are shown in Table 2. The basal diet (every 10 days) was sampled for the analysis of DM content (AOAC International, 2005; method 930.15) and CP (AOAC International, 2005; method 954.01) and starch (AOAC International, 2005; method 920.40) using the standard procedures of AOAC International [41]. The neutral detergent fiber and acid detergent fiber contents of the basal diet were determined as described by Van Soest et al. (1991) [42]. Zinc analysis was conducted using atomic absorption spectroscopy (Perkin Elmer atomic absorption spectrometer 3110, PerkinElmer, Waltham, MA). MAPRC (2004) feed ingredient tables were used for net energy, calcium and phosphorus, and the excel software was used for balancing the diet [40]. Clean and disinfect the cattle house before the test. All animals were housed in individual pens with free access to diets and water and under the constant care of a veterinarian. The cattle bed was 2 m long, and each cow was 1.2 m apart. The trough was 60 cm wide, 75 cm high and 25 cm deep. During the experiment, all individuals were fed at 6:00 am and 6:00 pm every day. The troughs were cleaned once each morning before feeding. Sinks were brushed every 7 days. Cowsheds were washed every 15 days. Sterilize every 30 days to prevent infectious disease.

**Table 2:** Ingredients and chemical composition of diet on a dry matter (DM) basis

	Content
Ingredient composition, %	
Corn grain	31.96
Wheat bran	28.12
Rapeseed meal	5.17
Distillers’ grains	10.00
Rice straw	20.00
Limestone	1.15
Salt	0.60
Sodium bicarbonate	2.00
Vitamin-mineral premixes^a^	1.00
Nutrition composition^b^	
Net energy, MJ/Kg	6.91
Crude protein, %	12.44
Neutral detergent fiber, %	32.07
Acid detergent fiber, %	21.13
Calcium, %	0.71
Phosphorus, %	0.72
Starch, %	36.83
Zn, mg/kg	29.22

^a^Vitamin-mineral premixes provided the following per kilogram diet: 1250 IU of vitamin A, 2000 IU of vitamin D3, 16 IU of vitamin E, 0.1 mg of Co, 9 mg of Cu, 0.6 mg of I, 54 mg of Mn, 0.2 mg of Se, and 33 mg of monensin

^b^Net energy, calcium and phosphorus were calculated values according to Feeding Standard of Beef Cattle (NY/T 815–2004), while others were measured values

From day 8 onwards (formal experiment), bulls were offered a diet every morning at 0600 h and 3 h ad libitum. The excess diet was collected and weighed daily for each bull. Feed ingredient and excess diet samples were collected every 10 days, and subsamples were dried at 55 °C for 48 h for determination of diet DM. Total DM intake (TDMI) was calculated as [(total feed offered × % DM) - (total feed refused × % DM)]/(number of animals × day) [43]. At the beginning and end of the feeding test, individually cattle were weighed on an empty stomach before morning feeding, recorded as the initial and final weights, the ADG was calculated, and finally, the feed efficiency was calculated as the ADG (kg/day) to TDMI (kg/day) ratio. After feeding for 60 days, 5 animals close to the average body weight within the group were selected for euthanasia and to collect samples. The cattle were euthanized with an intravenous injection of pentobarbital sodium (150 mg/kg body weight). Death was confirmed by auscultation for cardiac arrest. The middle colon mucosa tissue was collected by scraping the intestinal wall with glass microscope slides, frozen in liquid nitrogen and stored at − 80 °C for the analysis of intestinal cytokine and gene expression. TNF, IL-1β and IL-10 in colon mucosa were determined using a cattle ELISA kit (Nanjing Jiancheng Biochemical Reagent Co, Nanjing, China) according to the manufacturer’s instructions. Finally, colon digesta were rapidly frozen and stored at − 80 °C for microbiota population and BAs content analysis. The method of euthanasia was consistent with the recommendations of the Chinese Association for Laboratory Animal Sciences.

### Colonic digesta macrogenomic analysis

Colonic digesta bacterial DNA was extracted by a DNA Extraction Kit (Omega bio TEK, USA), and the quality of DNA was detected by 1% agarose gel electrophoresis. Then, the sample DNA was randomly broken into 400 bp fragments by a Covaris ultrasonic crusher, and the library was prepared by terminal repair, adding an A tail, adding a sequencing connector, purification and PCR amplification. A Qubit 2.0 fluorometer was used for preliminary quantification, and the library concentration was diluted to 2 ng/μL. The inserted fragments of the library were detected by an Agilent 2100 analyser. After the constructed library was tested to be qualified, Illumina PE2500 was sequenced according to the effective concentration.

The raw data obtained by sequencing were stored in FASTQ file format. The seqprep software was used to cut the adapter sequences at the 3′ and 5′ ends of the sequence. The reads with lengths less than 50 bp, average mass values less than 20 and N bases were removed by sickle software, and high-quality paired-end reads and single-end reads were retained. High-quality control data (clean data) were used for splicing, assembly and gene prediction to ensure the accuracy of the follow-up analysis results. After clustering with CD-HIT software (http://www.bioinformatics.org/cd-hit/). The longest gene in each class was taken as the representative sequence to construct a nonredundant gene set. The gene catalogue was translated into putative amino acid sequences, which were aligned against the proteins in NR (https://github.com/bbuchfink/diamond), evolutionary genealogy of genes: Nonsupervised Orthologous Groups (EggNOG, http://eggnog.embl.de/) and the Kyoto Encyclopedia of Genes and Genomes (KEGG, http://www.genome.jp/kegg/) databases with BLASTP (BLAST Version 2.2.28^+^, http://blast.ncbi.nlm.nih.gov/Blast.cgi).

### Colonic digesta BAs content analysis

Approximately 50 mg of the colonic digesta sample was weighed, and 400 mg/μL extract (methanol: water = 4:1) was added. The frozen tissue was ground for 6 min (− 10 °C, 50 Hz), ultrasonicated for 30 min (5 °C, 40 kHz) in a low-temperature ultrasound instrument, and allowed to stand at − 20 °C for 30 min. The samples were centrifuged for 15 min (4 °C, 13000 RCF), and 200 μL of supernatant was taken for machine detection. Standards for TCA (product number: 580217), TCDCA (product number: 700249P), GCDCA (product number: 700266P), CA (product number: C1129), GCA (product number: 700265P), ACA (product number: 700213P), TDCA (product number: T0875), DCA (product number: D2510), TLCA (product number: 700252P), β-MCA (product number: SML2372), 12-KLCA (product number: 700239P), apoCA (product number: 700241P), UDCA (product number: Y0001163), LCA (product number: BP927), isoLCA (product number: 700195P), DHLCA (product number: 700217P) and GDCA (product number: 700267P) were purchased from Sigma–Aldrich (Steinheim, Germany).

We weighed 1 mg of the standard, added methanol to a volume of 1 ml, vortexed and mixed evenly to obtain the standard stock solution. The LC-ESI-MS/MS (uhplc-qtrap 6500^+^) analysis method was used for qualitative and quantitative detection of the target substance in the sample. A Thermo Q exactive mass spectrometer (Thermo Fisher, UltiMate 3000 UHPLC) was used to collect primary and secondary mass spectrometry data. A Waters BEH C18 (100*2.1 mm, 1.7 μm) liquid chromatographic column was used with a column temperature of 40 °*C. mobile* phase A (0.1% formic acid aqueous solution) and mobile phase B (0.1% formic acid methanol). The inter- and intra-assay variation was controlled by limiting the coefficient of variation to ≤5% for all BAs variables. Finally, the content of BAs was calculated by a standard curve.

### Quantitative real-time PCR analysis

Total RNA of colon mucosa tissue was extracted by using TRIzol reagent. The concentration and quality of the total RNA were measured with a nucleic acid protein analyser. A 1% agarose gel was used for electrophoresis to detect its integrity. The RNA samples were reverse-transcribed by ImProm-II reverse transcriptase (Promega, Fitchburg, WI, USA) according to the kit instructions. According to the gene sequence published in GenBank, primers for real-time quantitative PCR of *the Bovine beta-actin* (*β-actin*), *GPBAR1*, *PRKACA* and *TNF* genes were designed. The *Bos taurus CREB1*, *IL-1β* and *IL-10* primers were designed by Wei et al. (2017) [44], Lahouassa et al. (2007) [45] and Leutenegger et al. (2000) [46], respectively. The gene primer sequences and amplification parameters are shown in Table 3. The real-time fluorescence quantitative PCR system was 20 μL: 2 μL cDNA, 10 μL SYBR Green 2 × Mix, 1 μL (100 nmol/L) upstream primer working solution, 1 μL (100 nmol/L) downstream primer working solution, and 6 μL ddH_2_O. The reaction conditions were as follows: predenaturation at 95 °C for 3 min, denaturation at 95 °C for 10 s, annealing for 30 s, and extension at 72 °C for 30 s for 40 cycles. This study determined the absence of contaminating genomic DNA and primer dimers in samples by analysing amplification and melting curves in negative controls consisting of DNase-treated total RNA without reverse transcriptase. *Β-actin* mRNA was used as the internal control for each sample, and the *Ct* value for each sample was normalized against that of *β-actin* mRNA. Target gene expression was calculated by the 2^-ΔΔ*Ct*^ method and relative to control samples.

**Table 3:** Specific primers used for real-time quantitative PCR

Gene	Primer	Accession
*β-actin*	F 5′-CAGCAAGCAGGAGTACGATG*-*3′	NM_173979.3
	R 5′-AGCCATGCCAATCTCATCTC*-*3’	
*GPBAR1*	F 5′-AGCATCCATCCATCTTGG*-*3’	Gene ID: 317756
	R 5′-GCTTTATTCAGTCAGAGTGGG*-*3’	
*PRKACA*	F 5′-GACCGAAGCCTGAGTGACAG-3’	Gene ID: 282322
	R 5′-CAGCTATGTACATCCTCGCG-3’	
*CREB1*	F 5′-GAGCCATTGATTTGTGCAAAGATG-3’	Wei et al. (2017)
	F 5′-GCGAGTGGTGAGAAGCGAAGTG-3’	
*TNF*	F 5′-AAGTAACAAGCCGGTAGCCCA*-*3’	NM_173966.3
	R 5′-CTTCCAGCTTCACACCGTTG*-*3’	
*IL-1β*	F 5′-CTCTCACAGGAAATGAACCGAG*-*3’	Lahouassa et al. (2007) [45]
	R 5′-CGCTGCAGGGTGGGCGTATCACC*-*3’	
*IL-10*	F 5′-CCAAGCCTTGTCGGAAATGA-3’	Leutenegger et al. (2000) [46]
	R 5′-GTTCACGTGCTCCTTGATGTCA-3’	

*GPBAR1* G protein-coupled bile acid receptor 1, *PRKACA* Protein kinase cAMP-activated catalytic subunit alpha, *CREB1* Cyclic-AMP response element binding protein 1, *TNF* Tumor necrosis factor, *IL-10* Interleukin-10, *IL-1β* Interleukin-1 β

### Statistical analyses

The growth performance, BAs content, cytokines and gene expression between the CON and NZnOC groups were analyzed by independent-samples *t* tests. Histograms were generated by GraphPad Prism 5. The Metagenomic data were analysed on the free online Majorbio I-Sanger Cloud Platform (www.i-sanger.com). To explore the dissimilarities in microbiota composition between the CON and NZnOC groups, PCoA based on the Bray–Curtis metric was performed at the phylum and genus level. Spearman’s correlations between secondary BAs content and secondary BAs synthesis bacterial genera were assessed by Origin 9.1. Spearman’s correlations between GPBAR1-PRKACA-CREB1 pathway marker gene expression and secondary BAs content were assessed by Origin 9.1. Data are shown as the means ± SD. Significance was declared at *p* < 0.05.

## Data Availability

The data shown in this paper are available within the article. The raw data of colonic digesta macrogenomic are available from the NCBI database under accession number PRJNA785327 (SUB10747183) https://www.ncbi.nlm.nih.gov/bioproject/785327.

## Abbreviations

BAs: Bile acid
CON: Control
NZnOC: Carbon loaded with nano-ZnO
BW: Body weight
TDMI: Total dry matter intake
ADG: Average daily gain
TCA: Taurocholic acid
TCDCA: Taurochenodeoxycholic acid
GCDCA: Glycochenodeoxycholic acid
CA: Cholic acid
GCA: Glycocholic acid
ACA: Allocholic acid
TDCA: Taurodeoxycholate acid
DCA: Deoxycholic acid
TLCA: Taurolithocholic acid
β-MCA: Beta-muricholic acid
12-KLCA: 12-ketolithocholic acid
apoCA: Apocholic acid
UDCA: Ursodeoxycholic acid
LCA: Lithocholic acid
isoLCA: Isolithocholic acid
DHLCA: Dehydrolithocholic acid
GDCA: Glycodeoxycholic acid
GPBAR1: G protein-coupled bile acid receptor 1
PRKACA: Protein kinase cAMP-activated catalytic subunit alpha
CREB1: Cyclic-AMP response element binding protein 1
TNF: Tumor necrosis factor
IL-1β: Interleukin-1 β
IL-10: Interleukin-10
